# Diabetes Mellitus Diagnosed in Childhood and Adolescence With Negative Autoimmunity: Results of Genetic Investigation

**DOI:** 10.3389/fendo.2022.894878

**Published:** 2022-06-13

**Authors:** Marilea Lezzi, Concetta Aloi, Alessandro Salina, Martina Fragola, Marta Bassi, Marina Francesca Strati, Giuseppe d’Annunzio, Nicola Minuto, Mohamad Maghnie

**Affiliations:** ^1^ Department of Neuroscience, Rehabilitation, Ophthalmology, Genetics, Maternal and Child Health, University of Genoa, Genoa, Italy; ^2^ Department of Pediatrics, IRCCS Istituto Giannina Gaslini, Genoa, Italy; ^3^ LABSIEM (Laboratory for the Study of Inborn Errors of Metabolism), IRCCS Istituto Giannina Gaslini, Genoa, Italy; ^4^ Department of Hematology and Oncology, Epidemiology and Biostatistics Section, IRCCS Istituto Giannina Gaslini, Genoa, Italy

**Keywords:** diabetes mellitus, monogenic diabetes, NEUROD1, INS (insulin), Next Generation Sequencing (NGS)

## Abstract

Monogenic diabetes is a rare form of diabetes, accounting for approximately 1% to 6% of pediatric diabetes patients. Some types of monogenic diabetes can be misdiagnosed as type 1 diabetes in children or adolescents because of similar clinical features. Identification of the correct etiology of diabetes is crucial for clinical, therapeutic, and prognostic issues. Our main objective was to determine the prevalence of monogenic diabetes in patients with diabetes mellitus, diagnosed in childhood or in adolescence, and negative autoimmunity. We retrospectively analyzed clinical data of 275 patients diagnosed with insulin-dependent diabetes at age <18yr in the last 10 years. 8.4% of subjects has negative autoimmunity. Their DNA was sequenced by NGS custom panel composed by 45 candidate genes involved in glucose metabolism disorder. Two novel heterozygous pathogenic or likely pathogenic variants (10,5% of autoantibody negative subjects) were detected: the frameshift variant c.617_618insA in NEUROD1 exon 2 and the missense change c.116T>C in INS exon 2. Our study corroborates previous results of other reports in literature. NGS assays are useful methods for a correct diagnosis of monogenic diabetes, even of rarest forms, highlighting mechanisms of pediatric diabetes pathogenesis.

## Introduction

Type 1 diabetes mellitus (T1DM) accounts for over 90% of children and adolescents with diabetes mellitus (1). It is a chronic, immune-mediated disease characterized by the destruction of insulin-producing β-cells in the pancreas. Over 90% of people with newly diagnosed type 1 diabetes have measurable serum antibodies against specific β-cell proteins, including insulin (IAA), glutamate decarboxylase (GADA), islet antigen 2 (IA2A), zinc transporter 8 (ZnT8A). The pathogenesis of T1DM is multifactorial: environmental factors interact with genetic susceptibility and the immune system, although the precise pathogenic processes underlying T1DM remain unclear (1).

Monogenic diabetes is a rare form of diabetes, accounting for approximately 1% to 6% of pediatric diabetes patients (2). It results from one or more defects in a single gene implicated in β-cell development or function. It may be inherited as a dominant, recessive, or non-Mendelian trait or may be due to a *de novo* mutation. The most common type of monogenic diabetes is Maturity Onset diabetes of the Young (MODY) and, to date, 14 subtypes are known (3).

About 10% of children and adolescents with insulin-dependent diabetes mellitus show no autoimmunity (4–6). Several reports in literature show that 6% to 12% of these patients have monogenic diabetes (4, 7–12). Indeed, some types of monogenic diabetes can be misdiagnosed as T1DM in children or adolescents because of similar clinical features (13, 14).

Identification of the correct etiology of diabetes is crucial for clinical management, therapeutic choices and prognosis, as well as for genetic counseling. Novel targeted next-generation sequencing (NGS) assay provides a highly sensitive method for simultaneous molecular analysis of several genes causative of monogenic diabetes, leading to a higher pathogenic variation detection rate, even of the most rare and uncommon forms (15, 16).

Our main aim was to determine the prevalence of monogenic diabetes in patients with insulin-dependent diabetes mellitus, diagnosed in childhood or in adolescence, with negative autoimmunity. Secondarily, we aim to determine any differences in clinical and/or biochemical characteristics at onset of diabetes that allow us to distinguish these two types of diabetes (autoimmune diabetes mellitus and negative-autoimmunity diabetes mellitus). We also aim to compare glycemic control between the two types of diabetes (autoimmune diabetes mellitus and negative-autoimmunity diabetes mellitus), considering HbA1c and insulin requirement during the first year after diagnosis at t1 (+6 months from onset) and t2 (+12 months from onset).

## Materials and Methods

We retrospectively analyzed data of 275 patients diagnosed with insulin-dependent overt diabetes at age <18 years from January 1st 2010 to and June 30th 2020, who underwent regular check-ups at the Regional Diabetes Center of the IRCCS Istituto Giannina Gaslini in Genoa, a tertiary care children’s hospital in Northern Italy. For each patient, anamnestic, clinical and biochemical data such as socio-demographic information, family history, comorbidities, onset parameters like HbA1c, serum bicarbonates, ketone bodies, the presence of diabetic ketoacidosis (defined as pH<7.3, serum bicarbonates <15mEq/L and ketone bodies >0.6mmol/L), Body Mass Index and autoimmunity status, were collected. Patients with mild/moderate hyperglycemia (not fulfilling criteria for diagnosis of diabetes and/or not requiring any therapy), secondary diabetes, psychiatric illness, or who were on drugs that affect blood sugar levels were excluded.

Positive autoimmunity was defined as the presence of at least one autoantibody against β-cells (IA2A, GADA, IAA, ZnT8A). Negative autoimmunity was defined as the absence of all tested autoantibodies. All patients (or parents if age < 18 years) provided a written informed consent in accordance with EU regulation 2016/679 to participate in the study. This study was approved by the local Ethical Committee.

### Genetic Tests

Multiplex primer pools were designed using Ion AmpliSeq Designer software (Thermo Fisher Scientific, MA, USA). This “on demand panel” is composed by 45 genes causative of dysglicemia and its complications (**Supplementary Table 1**) and it covers 100% of the coding region.

Genomic DNA was extracted from EDTA whole blood using QIAamp DNA Blood Midi kit (Qiagen GmbH, Hilden, Germany) and purified with Amicon Ultra 0,5 mL (Merk Millipore LTD, IRL). Eluted DNA was quantified with Nanodrop Spectrometer Thermo Fisher Scientific, MA, USA. Enrichment of exonic sequences was performed with an Ion AmpliSeq Library Kit 2.0 (Thermo Fisher Scientific, MA, USA) and sequenced on an Ion PGM (Thermo Fisher Scientific, MA, USA) using 316 Chip (Thermo Fisher Scientific, MA, USA) following the standard manufacturer’s protocol.

Sequencing data were analyzed with Coverage Analysis and Variant Caller plugins available within the Ion Torrent Suite software TS 5.18 and contextually with IonReporter. All the pathogenic or likely pathogenic variants detected by Ion PGM were amplified using specific couple of primers and confirmed by direct sequencing of the PCR products.

### Variant Interpretation

All the identified defects were checked for novelty utilizing free public HGMD (http://www.hgmd.cf.ac.uk/ac/index.php), gnomAD v2.1.1 (http://gnomad.broadinstitute.org/), dbSNP (https://www.ncbi.nlm.nih.gov/projects/SNP/) and Varsome (https://varsome.com/). The potential pathogenic role of all the identified variants was predicted by Mutation Taster (http://www.mutationtaster.org/), SIFT (http://www.blocks.fhcrc.org/sift/SIFT.html), PolyPhen-2 (http://www.genetics.bwh.harvard.edu/pph2). The pathogenicity of mutations was assessed in accordance with American College of Medical Genetics and Genomics (ACMG) guideline (17–19).

### Statistical Analysis

A descriptive statistic of the study population was performed. To assess the correlation between categorical variables, the Fisher test or the Chi-square test was used. The T-student test was used for the comparison between a categorical and a quantitative variable. P values ≤0.05 were considered statistically significant, and all P values were based on two tailed tests.

## Results

Two hundred seventy-five subjects (147 males and 128 females) with insulin-dependent diabetes (treated with Multiple Daily Injections, MDI, or Continuous Subcutaneous Insulin Infusion, CSII) diagnosed in childhood were included in the study. Negative autoimmunity was found in 23/275 patients (8.4%); consequently, participants were subdivided in two groups composed by 252 subjects with positive autoimmunity and 23 with negative autoimmunity, respectively. The main clinical characteristics, anamnestic and biochemical data of the participants subdivided in two subgroups are reported in **Table 1**. No significant differences were found in any of the items. Clinical data of all the 23 probands without β-cell antibodies are summarized in **Supplementary Table 2**.

**Table 1:** Clinical characteristics, anamnestic and biochemical data of the participants subdivided in 2 groups, expressed on average.

	Positive autoimmunity (n = 252)	Negative autoimmunity (n = 23)	p-value
Sex (%M:%F)	54:46	47.8:52.2	0.57
Average age at diagnosis (years)	8.68	8.99	0.74
DKA (%)	30.8	45.5	0.16
Symptoms (%)	93	89.5	0.64
Average duration of symptoms (years)	0.08	0.09	0.97
Average HbA1c (%) at diagnosis	11	10.5	0.37
Average c-peptide (ng/mL) at diagnosis	0.46	0.40	0.56
Average BMI at diagnosis (SDS)	0.19	-0.17	0.30
Family history of DM in first-degree relatives (%)	11.3	17.4	0.33
Associated autoimmune disease (%)	29	18.2	0.33
Average IDD** (U/kg) at t_1_/t_2_	0.5/0.62	0.49/0.58	0.89/0.44
Average HbA1c (%) at t_1_/t_2_	7.05/7.2	6.8/7.1	0.29/0.65

Statistical comparison between the two groups.

Molecular investigation of 19 out of 23 probands with absence of β-cell antibodies was requested and performed by NGS sequencing of 45 genes involved in blood glucose metabolism disorders and their complications. We could not perform the genetic test in 4 subjects of the original group because the written informed consent was not obtained.

In all the runs the mean coverage was over 200 pb. Genetic sequencing allowed us to detect two novel heterozygous pathogenic variants, causative of diabetes, in two unrelated probands: the frameshift variant c.617_618insA in Neurogenic Differentiation 1 gene (NEUROD1) exon 2 (patient 13) and the missense change c.116T>C in Insulin gene (INS) exon 2 (patient 19) (**Supplementary Table 3**).

NEUROD1 variant was found in a patient diagnosed with diabetes at 14 and half years, after coincidental findings of hyperglycemia. His mother had diabetes too. The patient was treated with only glargine insulin for 3 years, followed by insulin at meals because of worsening of glycemic profile. Recently, he started using an Advanced Hybrid Closed Loop (AHCL) technology with improvement of his glycemic profile. In early childhood, he was diagnosed with speech delay. The variant c.617_618 insA; p.His206Gln*38 in NEUROD1 [(OMIM: 601724) (Ref: NM_002500.5) (Ref: NP_002491)] inserts an adenine between nucleotides 617 and 618, creating an inactive truncated protein of 244 residues. To the best of our knowledge, this insertion has never been reported in literature and it is not present in the main databases such as gnomAD, 1000G, EXAC and dbSNP. The American College of Medical Genetics and Genomics (ACMG) guidelines classify it as “pathogenic”. Genetic analysis was extended to his parents, and their DNA was Sanger sequenced: the patient’s mother carried the same mutation, while the father has a wildtype genotype for the same allele (**Supplementary Figure 1A**).

INS mutation was found in a girl that was diagnosed with diabetes at 21 months, probably with DKA (there was no precise clinical information). Multiple dose insulin therapy was started. This patient showed a poor glycemic control, with HbA1c of about 10% and some DKA episodes. The missense change c. 116 T>C; p.Leu39Pro in INS (OMIM: 176730) (Ref: NM_000207.3) (Ref: NP_000198.1), leads to substitution of a high conserved leucine with proline at codon 39 (**Supplementary Figure 1B**). This variant has never been reported in literature and in the main allelic frequency databases (EXAC, gnomAD, 1000G, dbSNP). The “disease causing prediction tools “Mutation Taster, Sift and Poliphen2” classify it as “disease causing”, “damaging” and “probably damaging”, respectively. The ACMG guidelines classify it as “likely pathogenic”. The Leu39 residue is localized in α-helix domain, and the substitution with a proline disrupts the secondary protein structure, reducing the protein stability. DNA samples of the proband’s parents was not available, so a molecular investigation was not performed.

## Discussion

In our study, the molecular investigation allowed the identification of monogenic diabetes in 10.5% of patients with insulin-dependent overt diabetes mellitus diagnosed in childhood or adolescence and negative autoimmunity, that were 8.4% of our total cases. This data corroborates previous reports (4–6). In our cohort, no pathogenic variant of glucokinase (GCK) gene has been found, despite its major role in the pathogenesis of pediatric MODY, especially in patients from South Europe. We suppose that the clinical picture of overt diabetes, on which our data collection is centered, did not allow the identification of heterozygous mutations in the GCK gene generally associated with mild fasting hyperglycemia (20): this was an exclusion criteria in our sampling. Likewise, no pathogenic variant of HNF1A gene has been found, probably because our data collection is focused mainly on absent autoimmunity, not taking into account other MODY criteria, like family history of hyperglycemia before age 25 years among three consecutive generations.

NGS approach allowed us to detect two different heterozygous pathogenic variants in two unrelated probands: the frameshift insertion c.617_618insA in the exon 2 of NEUROD1 gene and the missense mutation c.116T>C in the exon 2 of INS gene. To the best of our knowledge, these variants have never been previously reported. These findings broaden the spectrum of mutations that cause monogenic diabetes in children.

In 1999, Malecki et al. firstly described two heterozygous NEUROD1 mutations in as many patients with type 2 diabetes (21). Over the next 20 years, NEUROD1 variants have been reported as causative of diabetes in almost 20 families (22). More recently, several other cases of MODY6 have been diagnosed all around the world, mainly due to widespread use of NGS technology (23–28). NEUROD1 is a transcription factor that is implicated in early development and differentiation of pancreatic α- and β-cells (29) and in activation of insulin gene transcription (30).

Biallelic NEUROD1 mutations have been described in patients with permanent neonatal diabetes mellitus and severe neurological manifestations (31, 32). Instead, heterozygous mutations had been linked to a rare (accounting less than 1% of cases) form of MODY, known as MODY 6 or NEUROD1-MODY (MIM # 606394), an autosomal dominant disease characterized by adolescent or adult-onset diabetes, with variable penetrance. Neurological manifestations are less frequent in individuals with NEUROD1-MODY, although developmental delay, minor cerebellar disfunction and cerebral atrophy had been described (33). Our NEUROD1-MODY patient showed a mild speech delay in early childhood, and this feature could be part of the clinical phenotype of the carried mutation, that is passed down from the maternal line like in other reports (22). The worsening of glycemic profile in our patient is likely due to a mechanism of glucotoxicity linked to NEUROD1 disfunction (22).

To the best of our knowledge, this is the first NEUROD1 mutation described in Italy in a pediatric patient. Recently, Brodosi et al. described a NEUROD1 variant in a 48-year-old patient diagnosed with MODY at the age of 25 (28). Making the right genetic diagnosis of MODY6 is important to start adequate treatment as soon as possible to prevent chronic hyperglycemia.

INS gene biallelic mutations are the second most common cause of permanent neonatal diabetes mellitus after the ATP-sensitive K+ (KATP) channels defects (34), instead INS monoallelic mutations are implicated in a rare MODY subtype (INS-MODY or MODY 10), (MIM # 613370) (35–38), especially in European countries (39–44). Insulin heterozygous pathogenic mutations lead to protein misfolding and subsequent β-cells oxidative stress. INS-related monogenic diabetes ranges from milder forms that benefit from diet to more severe clinical situations, even with ketoacidosis, with variable age of onset. Moreover, it has been shown that members of a same family with a same INS mutation may present a different clinical phenotype. Therefore, it has been proposed that environmental or epigenetic factors may contribute to pathogenesis of INS-MODY (38).

It is not known if our patient’s INS mutation is *de novo* or if it is inherited from one of her parents, because we did not perform genetic tests on them, and they do not suffer from blood glucose disorders. Nevertheless, considering the previously described phenotypic variability and the paternal family history of diabetes mellitus, it can be assumed that this mutation is inherited from the father who has not yet had clinical manifestations. This mutation is classified as pathogenic by the most used disease prediction tools and it determines a protein misfolding, so it’s likely that it is diabetes causing. Insulin is the best therapeutic choice or even better an insulin pump to improve glycemic control (40).

This study has some limitations. First of all, its retrospective nature results in lack of some clinical information. Moreover, it is monocentric, and the limited number of patients involved restricts the possibility of statistical analysis. In addition, the absence of statistical significance in the comparation of clinical and biochemical parameters between patients with positive autoimmunity and those with a negative one is due to the numeric difference of the two populations. On the other hand, our study provides new insights into pathogenesis of pediatric insulin-dependent overt diabetes, highlighting the importance of genetic tests in patients with negative autoimmunity. Molecular diagnosis is mandatory for characterization of diabetes, definition of the best therapeutic strategy and of prognosis, as well as for the risk of recurrence in first-degree relatives and future generations of affected patients.

Several reports in literature underline the relevance of NGS panels in diagnosis of monogenic diabetes, even of the rarest forms (27, 45–48). Although some clinical cases remain undiagnosed (13, 14), NGS provides rapid results increasing accuracy and reducing costs compared to Sanger sequencing (15). NGS panels can also be a useful screening method before performing more complex analyses, such as Whole Exome Sequencing (WES), which could also be more efficient in determining new genotype-phenotype associations.

As underlined by our study, the absence of ketoacidosis, considered a characteristic of T1DM, as well as of obesity, can no longer be considered as a peculiar feature of monogenic diabetes, since both have been described in patients with rare MODY types (49). A recent study has shown that the persistence of measurable C-peptide during follow-up, as well as the presence of positive family history for diabetes and a reduced insulin requirement (<0.5 IU/kg/day), are the best markers to distinguish MODY from T1DM (50). In a review from Jones et al., fasting C-peptide values >0.08 nmol/L (>0.24 ng/ml) after 3-5 years from diagnosis or values >0.4 nmol/L (> 1.2 ng/ml) at diagnosis are considered indicative of MODY (51). However, clinical and biochemical parameters are less frequently used to suspect monogenic diabetes. Our study supports this evidence, because the two patients diagnosed with monogenic diabetes have a very different clinical phenotype (**Table 2**). The only common feature is the absence of autoantibodies. Therefore, negative autoimmunity is confirmed to be the most specific parameter needed to start genetic analysis, as underlined by other studies (9).

**Table 2:** Clinical and biochemical characteristics of the 2 patients diagnosed with monogenic diabetes.

	Patient with NEUROD1-MODY	Patient with INS-MODY
Age at onset (yr)	14,8	1,8
DKA at onset	No	Yes
HbA1c at onset	6,6%	10,84%
C-peptide (ng/mL)	1,61	0,08
Auto-antibodies	Negative	Negative
IDD at last check-up (IU/kg/day)	0,36	1
History of diabetes in first-degree relatives	Yes (mother)	No

Our study results did not enable us to make substantial therapeutic changes, except the endorsement of addition of rapid-acting insulin analogue at meals in the NEUROD1-MODY patient to improve his glycemic control. Anyway, they are important for genetic counseling because both patients have a 50% chance of transmitting the disease to their children, as well as for the extension of genetic tests to first-degree relatives, or to other family members with hyperglycemia.

For the remaining 17 patients (about 6%) in our report with no molecular findings linked to monogenic diabetes, the most likely diagnosis is T1DM with negative auto-antibodies, also known as idiopathic T1DM, although we only have two of the four known antibodies available for most patients, therefore this may mask the real prevalence of autoimmune diabetes mellitus. Indeed, it has been shown that the assay of anti-ZnT8 antibodies increases the number of T1DM diagnosis, finding a positivity up to about 50% of patients with negativity of the remaining antibodies (52–54). In addition, other antigens such as tetraspanine-7 could be implicated in the auto-immune response in T1DM (55), although further studies are needed in order to better understand the structure of the self-antibody epitope. In addition, new genes have been associated with a clinical phenotype of MODY with low penetrance, such as RFX6 (tested in our panel), a transcription factor involved in the maturation and function of the β-cell (56, 57), and NKX6-1, also involved in the development of β-cells (58). Due to the limited number of previously reported cases and associated clinical information further studies are needed to define their genotype-phenotype correlation. However, mutations in unknown genes may account for some of the undiagnosed cases of diabetes mellitus and negative autoimmunity (59), so WES could be the next step for diagnosis.

In conclusion, monogenic diabetes is a rare form of pediatric diabetes. There aren’t useful clinical parameters to distinguish T1DM from monogenic diabetes. Negative autoimmunity remains the only tool that can guide genetic tests, thanks to its sensibility and specificity. NGS panels are a helpful technique that allows simultaneous analysis of several genes, leading to a correct molecular diagnosis, even of the rarest forms, and guiding therapy and prognosis. Whole exome sequencing could be used in patients with suspected T1DM and negative auto-antibodies.

## Data Availability Statement

The datasets presented in this article are not readily available because the genomic data are protected by privacy law. Requests to access the datasets should be directed to the corresponding author, nicolaminuto@gaslini.org.

## Ethics Statement

The studies involving human participants were reviewed and approved by CER Liguria. Written informed consent to participate in this study was provided by the participants’ legal guardian/next of kin. Written informed consent was obtained from the minor(s)’ legal guardian/next of kin for the publication of any potentially identifiable images or data included in this article.

## Author Contributions

Conceptualization, ML and NM. Methodology, CA, AS, and MF. Investigation, ML and MB. Resources, NM. Data curation, ML and MB. Writing—original draft preparation, ML, CA, and AS. Writing—review and editing, ML, MS, and NM. Visualization, NM. Supervision, Gd’A and MM. All authors have read and agreed to the published version of the manuscript.

## Conflict of Interest

The authors declare that the research was conducted in the absence of any commercial or financial relationships that could be construed as a potential conflict of interest.

## Publisher’s Note

All claims expressed in this article are solely those of the authors and do not necessarily represent those of their affiliated organizations, or those of the publisher, the editors and the reviewers. Any product that may be evaluated in this article, or claim that may be made by its manufacturer, is not guaranteed or endorsed by the publisher.
