# Supplementary material for: Diabetes Mellitus Diagnosed in Childhood and Adolescence With Negative Autoimmunity: Results of Genetic Investigation
Source: Front Endocrinol (Lausanne). 2022 Jun 13;13:894878. doi: 10.3389/fendo.2022.894878 (PMC9235348; doi:10.3389/fendo.2022.894878)
Supplement: Supplementary file 1 [file DataSheet_1.docx]

Supplementary Material

# Supplementary Figures and Tables

## Supplementary Figures

**Supplemetary Figure 1.** Pedigree and electropherogram of *NEUROD1* (A) and INS (B) mutations


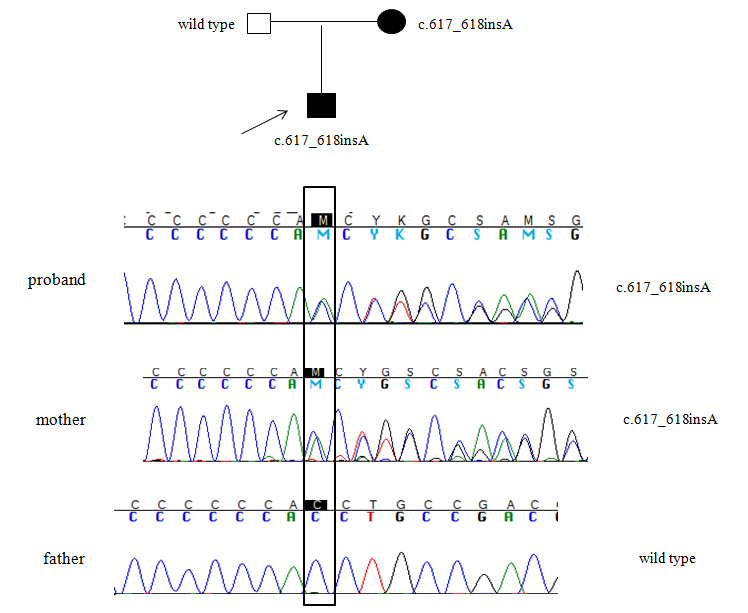
A)


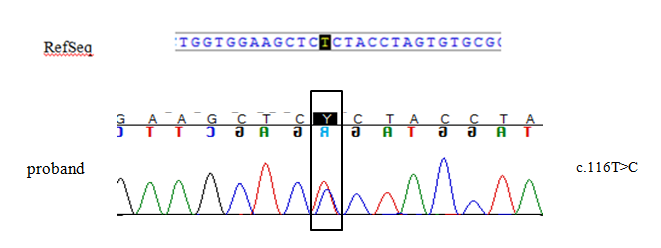
B)

## Supplementary Tables

**Supplementary Table 1: “**On demand panel” composed by 45 genes causative of dysglicemia and its complications.

| **Gene** | **Location** | **Phenotype** | **Phenotype MIM number** | **Inheritance** |
| --- | --- | --- | --- | --- |
| ABCC8 | 11p15.1 | MODY, type 12 | 600509 | AD, AR |
| AIRE | 21q22.3 | Autoimmune polyendocrinopathy syndrome, type I, with or without reversible metaphyseal dysplasia | 240300 | AD, AR |
| ALMS1 | 2p13.1 | Alstrom syndrome | 203800 | AR |
| APPL1 | 3p14.3 | {MODY, type 14} | 616511 | AD |
| AQP2 | 12q13.12 | Diabetes insipidus, nephrogenic, 2 | 125800 | AD, AR |
| AVPR2 | Xq28 | Diabetes insipidus, nephrogenic, 1 | 304800 | XLR |
| BBS1 | 11q13.2 | Bardet-Biedl syndrome 1 | 209900 | AR, DR |
| BLK | 8p23.1 | MODY, type 11 | 613375 | AD |
| CISD2 | 4q24 | Wolfram syndrome 2 | 604928 | AR |
| DIAPH1 | 5q31.3 | Deafness, autosomal dominant 1, with or without thrombocytopenia | 124900 | AD |
|  |  | Seizures, cortical blindness, microcephaly syndrome | 616632 | AR |
| GATA4 | 8p23.1 | Testicular anomalies with or without congenital heart disease | 615542 | AD |
|  |  | Atrial septal defect 2 | 607941 | AD |
|  |  | Atrioventricular septal defect 4 | 614430 | AD |
|  |  | Tetralogy of Fallot | 187500 | AD |
|  |  | Ventricular septal defect 1 | 614429 | AD |
| GATA6 | 18q11.2 | Pancreatic agenesis and congenital heart defects | 600001 | AD |
| GCK | 7p13 | MODY, type II | 125851 | AD |
| GJB2 | 13q12.11 | Deafness, autosomal recessive 1A | 220290 | AR, AD |
| GLIS3 | 9p24.2 | Diabetes mellitus, neonatal, with congenital hypothyroidism | 610199 | AR |
| GLUD1 | 10q23.2 | Hyperinsulinism-hyperammonemia syndrome | 606762 | AD |
| HADH | 4q25 | 3-hydroxyacyl-CoA dehydrogenase deficiency | 231530 | AR |
|  |  | Hyperinsulinemic hypoglycemia, familial, 4 | 609975 | AR |
| HNF1a | 12q24.31 | MODY, type III | 600496 | AD |
| HNF1b | 17q12 | MODY, type V Renal cysts and diabetes syndrome | 137920 | AD |
| HNF4a | 20q13.12 | MODY, type I | 125850 | AD |
| IL2RA | 10p15.1 | {Diabetes, mellitus, insulin-dependent, susceptibility to, 10} | 601942 |  |
|  |  | Immunodeficiency 41 with lymphoproliferation and autoimmunity | 606367 | AR |
| INS | 11p15.5 | MODY, type 10 | 613370 | AD |
| INSR | 19p13.2 | Hyperinsulinemic hypoglycemia, familial, 5 | 609968 | AD |
|  |  | Leprechaunism | 246200 | AR |
|  |  | Rabson-Mendenhall syndrome | 262190 | AR |
|  |  | Diabetes mellitus, insulin-resistant, with acanthosis nigricans | 610549 |  |
| KCNJ11 | 11p15.1 | MODY, type 13 | 616329 | AD |
| KFL11 | 2p25.1 | MODY, type VII | 610508 | AD |
| LRBA | 4q31.3 | Immunodeficiency, common variable, 8, with autoimmunity | 614700 | AR |
| MAGEL2 | 15q11.2 | Schaaf-Yang syndrome | 615547 | AD |
| NeuroD1 | 2q31.3 | MODY, type 6 Maturity-onset diabetes of the young 6 | 606394 | AD |
| OPA1 | 3q29 | Mitochondrial DNA depletion syndrome 14 (encephalocardiomyopathic type) | 616896 | AR |
|  |  | Behr syndrome | 210000 | AR |
|  |  | Optic atrophy 1 | 165500 | AD |
|  |  | Optic atrophy plus syndrome | 125250 | AD |
|  |  | {Glaucoma, normal tension, susceptibility to} | 606657 |  |
| OPA3 | 19q13.32 | 3-methylglutaconic aciduria, type III | 258501 | AR |
|  | 19q13.32 | Optic atrophy 3 with cataract | 165300 | AD |
| PAX4 | 7q32.1 | MODY, type IX | 612225 | AD |
| PAX6 | 11p13 | Coloboma of optic nerve | 120430 | AD |
|  |  | Coloboma, ocular | 120200 | AD |
|  |  | Morning glory disc anomaly | 120430 | AD |
|  |  | Aniridia | 106210 | AD |
|  |  | Anterior segment dysgenesis 5, multiple subtypes | 604229 | AD |
|  |  | Cataract with late-onset corneal dystrophy | 106210 | AD |
|  |  | Foveal hypoplasia 1 | 136520 | AD |
|  |  | Keratitis | 148190 | AD |
|  |  | Optic nerve hypoplasia | 165550 | AD |
| PDX1/IPF1 | 13q12.2 | MODY, type IV | 606392 | AD |
|  |  | Pancreatic agenesis 1 | 260370 | AR |
|  |  | {Diabetes mellitus, type II, susceptibility to} | 125853 | AD |
| POU3F4 | Xq21.1 | Deafness, X-linked 2 | 304400 | XLR |
| RFX6 | 6q22.1 | Mitchell-Riley syndrome | 615710 | AR |
| SEL1L | 14q31.1 | branchial cleft syndrome involving hypertelorism, preauricular sinus, punctal pits, and deafness, | 614187 | AD, AR |
| SH2B1 | 16p11.2 | severe obesity, insulin resistance, and neurobehavioral abnormalities | 608937 | AD |
| SLC5A2 | 16p11.2 | Renal glucosuria | 233100 | AD, AR |
| SOX9 | 17q24.3 | Acampomelic campomelic dysplasia | 114290 | AD |
| SOX17 | 8q11.23 | Vesicoureteral reflux 3 | 613674 | AD |
| STAT1 | 2q32.2 | Immunodeficiency 31A, mycobacteriosis, autosomal dominant | 614892 | AD |
|  |  | Immunodeficiency 31B, mycobacterial and viral infections, autosomal recessive | 613796 | AR |
|  |  | Immunodeficiency 31C, chronic mucocutaneous candidiasis, autosomal dominant | 614162 | AD |
| STAT3 | 17q21.2 | Autoimmune disease, multisystem, infantile-onset, 1 | 615952 | AD |
|  |  | Hyper-IgE recurrent infection syndrome | 147060 | AD |
| STAT5B | 17q21.2 | Growth hormone insensitivity with immune dysregulation 1, autosomal recessive | 245590 | AR |
|  |  | Growth hormone insensitivity with immune dysregulation 2, autosomal dominant | 618985 | AD |
| TMEM126 |  | Optic atrophy 7 | 612989 | AR |
| WFS1 | 4p16.1 | Wolfram Syndrome 1 | 222300 | AR |

# Supplementary Table 2: Anamnestic, clinical and biochemical characteristics of patients with negative autoimmunity. NA: not available. DM: diabetes mellitus. DKA: diabetic ketoacidosis. BMI: Body Mass Index. IDD: Insulin Daily Dose. ASD: Atrial Septal Defects. VSD: Ventricular Septal Defects. T: Hashimoto’s thyroiditis. C: Coeliac disease. *Gestational Diabetes Mellitus

| **Patient** | **Gender** | **Ethnicity** | **Age at diagnosis (yr)** | **DKA** | **HbA1c at diagnosis (%)** | **C-peptide (ng/mL)** | **BMI (kg/m^2^)** | **Comorbidity** | **GADA** | **IAA** | **IA2A** | **ZnT8A** | **Family history of DM** | **Other auto-immunity** | **IDD (IU/kg) t_1_** | **IDD (IU/kg) t_2_** | **HbA1c (%) t_1_** | **HbA1c (%) t_2_** |
| --- | --- | --- | --- | --- | --- | --- | --- | --- | --- | --- | --- | --- | --- | --- | --- | --- | --- | --- |
| 1 | M | Caucasian | 7,1 | Yes | 11,5 | 0,65 | 19,0 | Hypospadias, cryptorchidism, mental retardation | neg | neg | neg | neg | / | No | 0,66 | 0,7 | 6,3 | 7,2 |
| 2 | F | Caucasian | 6,1 | Yes | 9,2 | 0,4 | 14,3 | ASD, VSD | neg | neg | NA | NA | / | No | 0,44 | 1,1 | 7,7 | 8,3 |
| 3 | F | Caucasian | 10,2 | No | NA | 0,1 | NA | Asthma | neg | neg | NA | NA | / | T/C | NA | NA | 5,8 | 7,1 |
| 4 | F | Caucasian | 6,0 | No | 9,1 | 0,3 | NA | Astigmatism, hyperopia, macrohematuria | neg | neg | NA | NA | / | No | 0,35 | 0,42 | 6,7 | 6,7 |
| 5 | M | Caucasian | 7,9 | No | 12 | 0,18 | 14,3 | / | neg | neg | NA | NA | / | No | 0,5 | 0,48 | 7 | 7,7 |
| 6 | M | Caucasian | 9,6 | Yes | 12,8 | 0,1 | NA | / | neg | neg | NA | NA | / | No | 0,41 | 0,5 | 8,2 | 7,7 |
| 7 | M | Caucasian | 13,6 | No | 7,62 | 0,5 | 14,9 | Behavioural problems | neg | neg | neg | neg | Father | No | 0,24 | 0,4 | 6,1 | 6,8 |
| 8 | F | Caucasian | 9,3 | Yes | 12,2 | NA | NA | / | neg | neg | neg | NA | / | No | 0,5 | 0,53 | 7,5 | 6,2 |
| 9 | F | Caucasian | 10,9 | No | 12,1 | NA | NA | / | neg | neg | neg | NA | / | No | 0,3 | 0,33 | 6,9 | 7,4 |
| 10 | M | Caucasian | 13,5 | No | 11,27 | 0,2 | 19,9 | / | neg | neg | NA | NA | / | C | 0,2 | 0,5 | 6,3 | 7,8 |
| 11 | M | Caucasian | 16,1 | No | 13,54 | NA | 17,6 | / | neg | neg | neg | neg | / | No | 0,8 | 0,6 | NA | 7,7 |
| 12 | F | African | 5,7 | Yes | NA | 0,2 | NA | Mental retardation, polymicrogyria (WDR62 mutation) | neg | neg | NA | NA | Father, mother* | No | 0,5 | 0,5 | 6,3 | 6,8 |
| 13 | M | Caucasian | 14,8 | No | 6,2 | 1,61 | 19,4 | Hydrocele, delayed speech | neg | neg | neg | neg | Mother | No | 0,2 | 0,2 | 5,6 | 5,5 |
| 14 | F | Caucasian | 9,6 | Yes | 12,69 | 0,42 | 18,6 | Central hypothyroidism | neg | neg | NA | NA | / | No | 1 | 1,1 | 8,8 | 7,8 |
| 15 | M | Caucasian | 12,8 | Yes | 15,56 | 0,1 | 18,1 | / | neg | neg | NA | NA | / | No | 0,5 | 0,55 | 6,7 | 7,1 |
| 16 | F | Caucasian | 4,6 | No | 9,7 | NA | NA | / | neg | neg | NA | NA | / | No | NA | NA | NA | NA |
| 17 | M | Caucasian | 5,9 | No | 10,2 | NA | 15,0 | / | neg | neg | NA | NA | / | No | NA | 0,6 | 6,9 | 7,2 |
| 18 | F | Caucasian | 7,3 | Yes | 10,31 | NA | 14,1 | / | neg | neg | NA | NA | / | No | 0,74 | 0,71 | 7,1 | 7,1 |
| 19 | F | African | 1,8 | NA | NA | NA | NA | / | neg | NA | neg | NA | / | No | NA | NA | NA | NA |
| 20 | M | Caucasian | 11,1 | Yes | 12,3 | NA | 16,9 | / | neg | neg | NA | NA | / | No | 0,36 | 0,47 | 6,9 | 7,1 |
| 21 | F | African | 5,2 | Yes | 10,79 | 0,4 | 14,6 | / | neg | neg | NA | NA | / | No | 0,65 | 0,75 | 6,4 | 6,5 |
| 22 | F | Caucasian | 6,8 | No | NA | NA | NA | / | neg | neg | neg | NA | / | T | NA | NA | NA | NA |
| 23 | M | Caucasian | 11,0 | No | 9,83 | NA | 17,5 | / | neg | neg | NA | NA | Mother | T/C | 0,4 | 0,5 | 5,9 | 6,4 |

**Supplementary Table 3.** Variants found through "NGS Panel" (45 genes) in patients 13 and 19. Pt: Patient. DC: Disease Causing, D: Damaging, PD: Probabily Damaging, ND: Not determined

| **Pt** | **Gene** | **Nucleotide change / Exon** | **Aminoacidic change** | **ACGM Classification** | **Disease causing prediction tools** | | | **Ref.** |
| --- | --- | --- | --- | --- | --- | --- | --- | --- |
|  |  |  |  |  | **Mutation Taster** | **SIFT** | **Polyphen 2** |  |
| 13 | NEUROD1 (NM_002500.5) | c.617_618 insA / 2 | p.His206Glnfs Ter38 | Pathogenic | ND | ND | ND | This study |
| 19 | INS (NM_000207.3) | c.116T>C / 2 | p.Leu39Pro | Likely Pathogenic | DC | D | PD | This study |
